# Histone H3K4me3 and H3K27me3 regulatory genes control stable transmission of an epimutation in rice

**DOI:** 10.1038/srep13251

**Published:** 2015-08-19

**Authors:** Xiangsong Chen, Xiaoyun Liu, Yu Zhao, Dao-Xiu Zhou

**Affiliations:** 1 National Key Laboratory of Crop Genetic Improvement, Huazhong Agricultural University, 430070 Wuhan, China; 2 Institute for Interdisciplinary Scientific Research, Jianghan University, 430056 Wuhan, China; 3 Institute Plant Science Paris-Saclay (IPS2), Université Paris-sud 11, 91405 Orsay, France

## Abstract

DNA methylation loss can produce inheritable active epialleles in plants. The mechanism involved in the stable transmission of hypomethylated epimuations is presently not clear. Here we show that maintenance of a stably hypomethylated active epiallele in rice required a CHD3 protein (CHR729) and that over-expression of an H3K4me3 demethylase (JMJ703) or H3K27me3 methyltransferase (SDG711) could stably resilence the epiallele. CHR729 and JMJ703 have antagonistic function in H3K4me3 in maintaining the active state of the epiallele, whereas SDG711-mediated H3K27me3 was sufficient to stably repress the locus. The data suggest that H3K4me3 and H3K27me3 controlled by these chromatin regulators may be involved in stable transmission/resetting of epigenetic variation in rice.

Epigenetic processes can stably alter gene transcriptional activities independently of the DNA sequence. Several biochemical mechanisms that lead to changes in DNA methylation and/or histone modifications have been discovered to be involved in the processes^1^. DNA methylation is important for diverse epigenetic phenomena in plants, including repression of transposable elements (TEs) and repetitive sequences and genomic imprinting.

In plants, cytosine methylation is found in three sequence contexts: CG, CHG, and CHH (in which H = A, T, C). The DOMAINS REARRANGED METHYLTRANSFERASE2 (DRM2) catalyzes *de novo* DNA methylation in all sequence contexts and maintains asymmetric CHH methylation. Small RNAs target DRM2 to homologous genomic DNA sequences for cytosine methylation by the RNA-directed DNA methylation (RdDM) pathway. Symmetric CG methylation is maintained by METHYLTRANSFERASE1 (MET1), which recognizes hemimethylated CG sites after DNA replication. CHG methylation is catalyzed by the plant specific CHROMOMETHYLASE3 (CMT3). Other genes such as the histone deacetylase HDA6, and the SNF2/SWI2 chromatin remodeler DDM1 (decrease in DNA methylation 1) are also involved in DNA methylation^2^. Defects in DNA methylation genes such as *met1* and *ddm1* mutations lead to genome-wide hypomethylation^3^. DNA methylation loss from transposons in *ddm1* mutants can be recovered by RdDM, when wild type *DDM1* is reintroduced^4^. However there are many hypomethylated genomic regions that cannot be recovered, as heritable hypomethylated chromosomal segments have been propagated for many generations in so called “epiRILs” (epigenetic recombinant inbred lines)^5^. In *ddm1* and *met1* mutants hypomethylated TEs neighboring genes resulted in stable epimutations such as *BONSAI* and *FWA* (*FLOWERING WAGENINGEN*)^3^.

The rice genomic DNA is methylated also in all three cytosine contexts, with high levels of CG and CHG methylation and very low levels of CHH methylation^6^,^7^. Genes involved in DNA methylation are generally conserved in rice^8^,^9^. Besides defects in DNA methylation genes, plant tissue-culture also leads to general DNA methylation loss^10^,^11^. Loss of DNA methylation during rice callus culture was found to be associated with loss of 24 nt siRNA in callus-regenerated plants and was largely stable across generations^11^. However, the mechanism that maintains the transgenerationally stably hypomethylated state is not known.

Besides DNA methylation, histone modifications and chromatin remodeling also play important roles in epigenetic regulation of gene expression. For instance, trimethylation of Histone H3 lysine 4 (H3K4me3) is associated with gene activation, whereas trimethylation of Histone H3 lysine 27 (H3K27me3) marks repressed genes. Plant SET-domain genes (SDG) are involved in histone methylation that can be removed by specific histone demethylases such as Jumonji (JMJ)-C domain containing proteins identified in rice^8^,^12^.

In this work we characterized a stably hypomethylated and transcriptionally active locus in rice callus regenerated plants. We show that the stable hypomethylation and activation of the locus required the CHD3 chromatin remodeling factor CHR729^13^, which is involved in maintenance of H3K4me3 during cell differentiation. Removal of H3K4me3 by histone demethylase JMJ703^14^,^15^, resulted in repression and recovery of DNA methylation of the locus. Our data reveals that H3K4me3 was involved in the transmission of the active state of the epiallele and that, in addition to DNA methylation, H3K27me3 also played a role in transgenerationally stable gene repression in rice.

## Results and Discussion

### Identification of a callus culture-induced stable epimutation in rice

**Figure 1:**
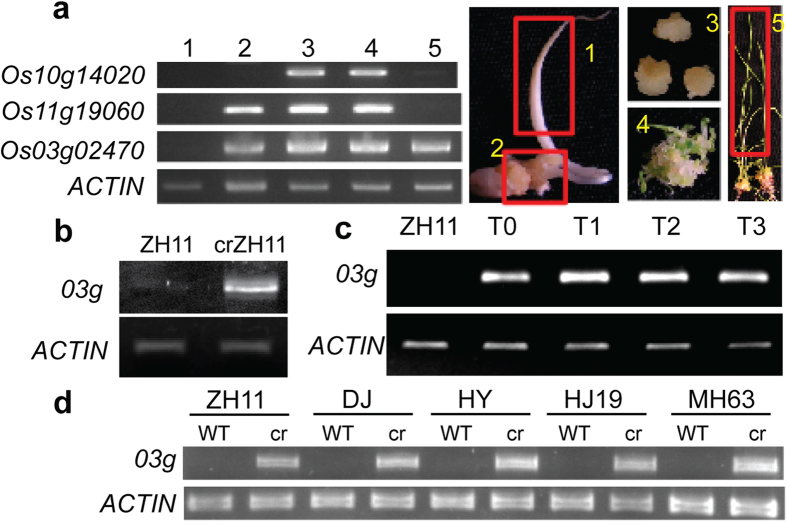
Callus culture induced expression of Os03g02470 (*03g*) was stably inherited. (**a**) RT-PCR detection of transcripts of three callus-specific genes during callus culture and plant regeneration processes. The stages of callus culture and regeneration are shown in the right panel. 1: seed germinated; 2: callus-like tissue in the early stage of callus induction; 3: subcultured callus; 4: mixture of callus and shoot during regeneration stage; 5: regenerated shoots/leaves. (**b**) *03g* expression is detectable in pollens of callus-regenerated crZH11 compared to normal ZH11 pollens. (**c**) *03g* expression was maintained in 4 subsequent generations. mRNA isolated from leaves of T0 to T3 generation plants were analyzed. (**d**) *03g* expression was detected in all independent callus regeneration (cr) plants of different rice varieties as indicated. PCR products of different samples for each primer set were run in the same gel and cropped in the same picture.

**Figure 2:**
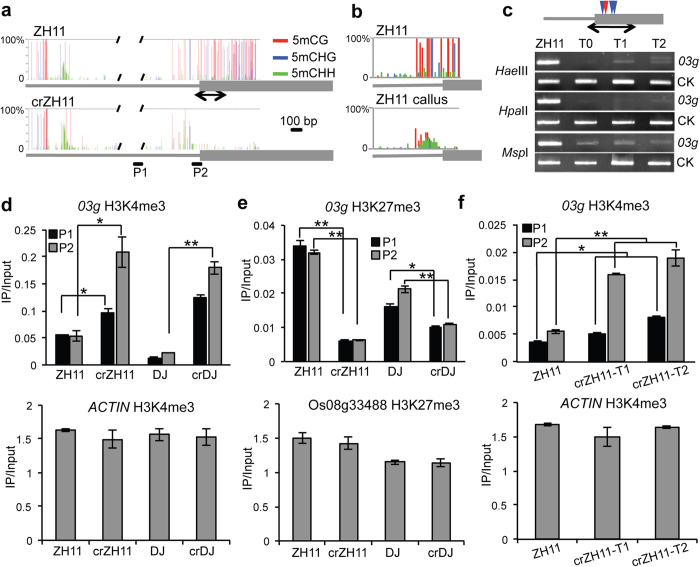
Stable *03g* activation was associated with DNA methylation loss, histone H3K27me3 removal and H3K4me3 deposition. (**a**) *03g* was heavily methylated in CG and CHG contexts at transcription start site (TSS) and 5′ UTR in leaf tissues of normal wild type ZH11, but demethylated in leaf tissues of callus regenerated ZH11 (crZH11) plants. DNA methylation was determined by bisulfite sequencing. More than 20 clones for each genotype were sequenced. Solid gray line: upstream promoter region; grey bar: transcribed region of *03g* gene. Primers sets (P1, P2) used in ChIP assays are indicated. Black slashes indicate the gap, which is not sequenced. (**b**) DNA methylation of *03g* was lost in callus. Bisulfite sequencing region is represented by the double arrows shown in a. (**c**) DNA demethylation was maintained in subsequent generations, shown by enzyme digestion followed by PCR amplification. PCR amplification region was the same as bisulfite sequencing region in a. CK, control DNA fragments without enzyme recognition sites. Blue triangle indicates *Hpa*II/*Msp*I cut sites, red triangle indicates *Hae*III cut sites. PCR products of different samples for each primer set were run in the same gel and cropped in the same picture. (**d**,**e**) H3K4me3 (**d**) and H3K27me3 (**e**) at promoter (P1) and TSS region (P2) of *03g* were respectively increased and decreased in callus-regenerated (cr) plants of two different rice cultivars (ZH11 and DJ). (**f**) Increased H3K4me3 in *03g* was maintained in subsequent T1 and T2 generations. *ACTIN* was used as a control for H3K4me3. Os08g33488, which is methylated by H3K27me3^15^, was used as a control for H3K27me3. Student *t-*tests were performed from 3 biological repeats. *p < 0.05, **p < 0.01.

Previous data have shown that many genes are activated during callus culture^16^. However, most of the callus culture-induced genes are expressed in different developmental stages and/or organs/tissues and only a few genes display callus-specific expression^16^. Analysis by RT-PCR revealed that these genes that are expressed only in callus were resilenced during plant regeneration process, except *Os03g02470* (referred as to *03g* thereafter) that was not repressed in regenerated plant leaves (Fig. 1a). Transcripts of the gene could be detected in pollen and the active state of the locus was maintained in subsequent generations (Figs 1b,c). The active state of the locus was detected in all examined regenerated plants from independent callus cultures of different rice varieties (Fig. 1d), suggesting that callus culture-induced activation of the gene was not stochastic. The *03g* locus encodes a protein of unknown function. In leaf tissues of normal plants, the promoter and the 5′ region of the gene displayed heavy CG (>90%) and CHG (H = A, T, or C) (>40%) methylation, which were lost in callus and regenerated shoots (Fig. 2a,b). While the other two loci, Os10g14020 and Os11g19060, were not methylated either in leaf or callus. The hypomethylation state of *03g* was maintained in subsequent generations (Fig. 2c). Recent results have identified many genes that show DNA methylation loss during rice callus culture and stable hypomethylation in regenerated plants^11^. Hypomethylation was also found in the *03g* locus in that study, but it was attributed to the downstream gene (*Os03g02460*), the promoter of which actually overlapped with *03g*. H3K4me3 was increased, and H3K27me3 was decreased in the locus in regenerated plants (Fig. 2d,e). By comparison, H3K4me3 levels were not clearly changed in Os10g14020 and Os11g19060, although a reduction of H3K27me3 was detected in Os10g14020 (Supplementary Figure 1). In addition, the increased H3K4me3 levels in *03g* were maintained in the subsequent generations (Fig. 2f). The activation of *03g* was likely to be triggered by loss of DNA methylation during callus culture, as treatment of normal ZH11 seedlings with 5-azacytidine (an inhibitor of DNA methylation) led to DNA hypomethylation, gene expression, and increased H3K4me3 of the locus (Supplementary Figure 2). Moreover, the DNA hypomethylation and gene activation were maintained in the subsequent generation (Supplementary Figure 2).

To test whether the hypermethylated *03g* allele in normal plants could influence the hypomethylated allele for expression, we crossed callus-regenerated ZH11 (crZH11) with normal MH63 plants, in which varieties *03g* is polymorphic (Supplementary Figure 3a). *03g* was found to be expressed in all tested F1 individuals and all of the sequenced transcripts were derived from the ZH11 allele (Supplementary Figure 3a). In addition, in crosses between crZH11 and normal ZH11 plants, about ¾ of the F2 segregates displayed *03g* expression (Supplementary Figure 3b), suggesting that the hypomethylated epiallele was not controlled by the hypermethylated allele or vice versa.

### Histone methylation regulators are involved in the maintenance of stable expression of the epiallele

**Figure 3:**
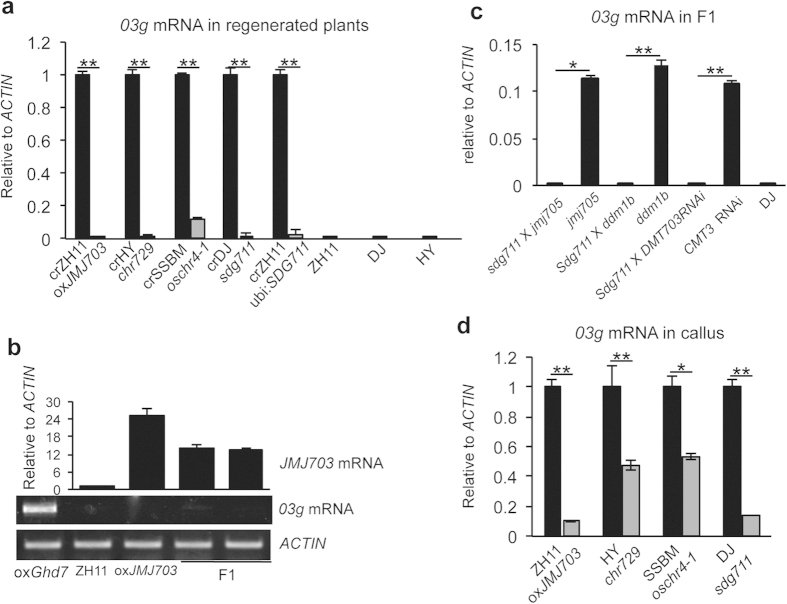
Callus culture-induced *03g* expression was repressed by over-expression of *JMJ703* and *SDG711 and* mutations of *CHR729*. (**a**) *03g* expression was repressed in *JMJ703* over-expression (*oxJMJ703*), *SDG711* gain-of-function T-DNA mutant (*sdg711*) and over-expression (*ubi: SDG711*), and *CHR729* T-DNA (*chr729*) and EMS-induced (*oschr4*) mutant plants compared to the respective regenerated wild plants (crZH11, crHY, crDJ, and crSSBM). The expression levels are presented as relative to regenerated wild type plants (set at 1). (**b**) *03g* expression was inhibited in the F1 hybrids of crossed between *oxJMJ703* and a *03g-expressing* transgenic plant (*oxGhd7*). (**c**) *03g* expression was inhibited in the F1 hybrids of crosses between *sdg711* and other three *03g* active regenerated plants (i.e. T-DNA mutants of *jmj705* and *ddm1b*, and *DMT703* RNAi). *03g* expression levels were normalized with *ACTIN.* (**d**) *03g* expression was detected in callus of *oxJMJ703, chr729, oschr4, sdg711,* and *ubi: SDG711* plants. But the expression levels were reduced compared to the respective wild types (set at 1). Student *t-*tests were performed from 3 biological repeats. *p < 0.05, **p < 0.01.

To study whether the stable activation of *03g* could be affected by loss- or gain-of-function of DNA methylation, histone modification, and other chromatin regulatory genes, we analyzed *03g* expression in rice T-DNA mutants, RNAi and over-expression transgenic plants of 16 chromatin protein genes encoding DNA methyltransferases (*DMT702*, *DMT703, DMT706,* and *DMT707*) (Supplementary Figure 4), DNA demethylase (*DNG701*)^17^, SET-domain histone methyltransferases (*SDG711, SDG721*, *SDG723* and *SDG728*) (Supplementary Figure 4)^14^,^18^,^19^,^20^, Jumonji-C histone demethylases (*JMJ703*, *JMJ705*, *JMJ706* and *JMJ716*) (Supplementary Figure 4)^21^,^22^,^23^,^24^, chromatin remodeling factors involved in DNA methylation (*DDM1*)^25^, histone modification (*CHR729*)^13^, and recognition (*LHP1*) (Table 1 and Supplementary Figure 4). The tissue culture-induced *03g* activation was detected in most of the T-DNA mutants and transgenic plants, except in *CHR729* loss-of-function T-DNA mutant (*chr729*)^13^, *JMJ703* over-expression lines (ox*JMJ703*)^21^,^22^ and a gain-of-function T-DNA insertion mutant of *SDG711* (*sdg711*)^14^,^15^, in which *03g* was silenced as in normal wild type plants (Table 1 and Fig. 3a). The down-regulation of *03g* expression in the *chr729* T-DNA line was confirmed in callus-regenerated plants of an ethyl methanesulfonate (EMS)-induced mutant allele of *chr729,* known as *oschr4-1*^26^ (Fig. 3a). In independent *SDG711* over-expression lines (under the control of the maize ubiquitin gene promoter*, Ubi: SDG711)*^14^ , *03g* was also repressed (Table 1 and Fig. 3a). To further confirm whether increased levels of JMJ703 repressed *03g* expression, we crossed a transgenic plant (i.e. *OxGhd7*, which is MH63 background with overexpression of rice flowering regulator *Ghd7* and showed active *03g* expression)^27^,^28^ with the ox*JMJ703* line and found that the *03g* expression was repressed in the F1 hybrids (Fig. 3b), while remained to be expressed when crossed with normal plants (Supplementary Figure 2). Similarly, *03g* expression was resilenced in the F1 hybrids of crosses between *sdg711* and three different transgenic plants (Fig. 3c). These data indicated that over-expression of *JMJ703* or *SDG711* suppressed *03g* expression. *JMJ703* encodes a histone H3K4me3 demethylase^21^,^22^, and *SDG711,* a homolog of Enhancer of zeste, encodes a histone H3K27me3 methyltransferase^14^,^18^. Thus, in addition to DNA methylation, histone methylation homeostasis regulated by CHR729, JMJ703 and SDG711 could also control stable expression state of the gene.

To check whether the repression of *03g* expression in the above mentioned mutants and transgenic plants was initiated early during callus induction or shoot regeneration process, we produced callus from *chr729*, *oxJMJ703*, and *sdg711* plants and found that unlike in the respective regenerated plants where *03g* was almost totally silent, the transcripts of *03g* could be detected in callus derived from these plants especially from *chr729* and *oschr4-1* mutants (Fig. 3d), suggesting that repression of *03g* by *CHR729* mutation or *SDG711* and *JMJ703* over-expression initiated during tissue culture was reinforced during plant regeneration.

### Antagonistic function of CHR729 and JMJ703 is involved in regulating the *03g* expression state

**Figure 4:**
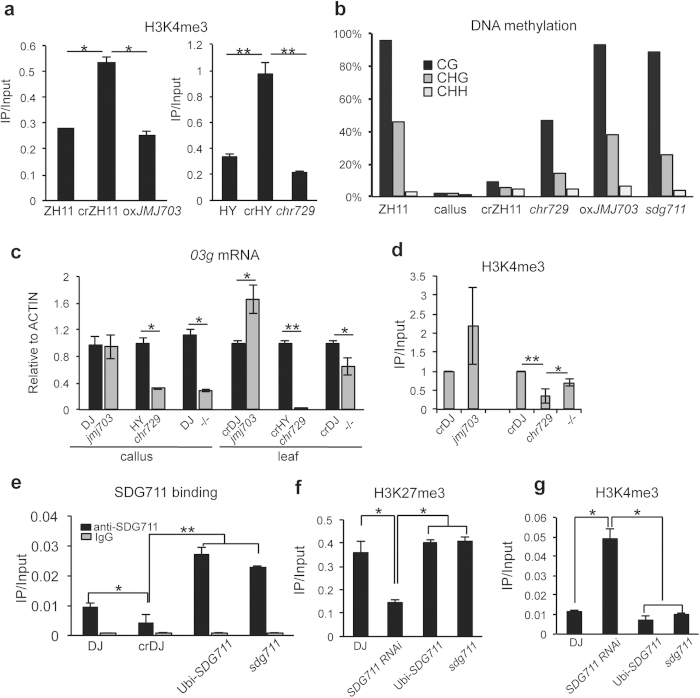
H3K4me3 and H3K27me3 had antagonistic functions in stabilizing the expression and DNA methylation levels on *03g* locus. (**a**) H3K4me3 in *03g* was reduced in *oxJMJ703* and *chr729* mutant respectively compared to in wild type regenerated plants. (**b**) DNA methylation of *03g* locus was partially or fully recovered in *chr729* mutant, *oxJMJ703,* and *sdg711* plants. DNA methylation was determined by bisulfite sequencing. More than 20 clones for each genotype were sequenced. (**c**) Comparison of *03g* expression in *jmj703*, *chr729,* and *jmj703*/*chr729* double mutant (−/−) callus and leaves compared to wild type (DJ, HY) or regenerated wild type leaves (crDJ, crHY). The expression levels were normalized with *ACTIN* and then set as 1 in wild types. (**d**) H3K4me3 levels of *03g* in *jmj703, chr729,* and *jmj703/chr729* double mutant (−/−). (**e**) SDG711 protein was enriched in *03g*. ChIP analysis was performed with anti-SDG711^14^ and with preimmune serum (IgG) as controls. (**f**,**g**) H3K4me3 (**f**) and H3K27me3 (**g**) levels of *03g* in *SDG711* RNAi, over-expression (*ubi: SDG711*), and gain-of-function T-DNA line (*sdg711*) compared to wild type (DJ). Because H3K4me3 and H3K27me3 are enriched in the 5′ end of pant genes, P2 primer set shown in Fig. 2a was used for the ChIP analysis. Primers used for ChIP qPCR were P2 indicated in Fig. 2a. Student *t-*tests were performed from 3 biological repeats. *p < 0.05, **p < 0.01.

CHR729 was shown to be preferentially involved in regulating H3K4me3 levels of under-expressed genes^13^. The requirement of *CHR729* for the maintenance of *03g* expression during plant regeneration and the opposite effect of *JMJ703* over-expression suggests that H3K4me3 levels may be important for the stabilization of the callus-induced epigenetic expression state of the gene. Analysis of chromatin modifications on the *03g* locus revealed a decrease of H3K4me3 (Fig. 4a) and restoration of DNA methylation in *chd729* T-DNA mutant and *JMJ703* over-expression plants compared to regenerated wild type (Fig. 4b). However, in *jmj703* loss-of-function mutant^21^,^22^, *03g* expression in callus was not affected, while some increase of *03g* expression was observed in *jmj703* leaves (Fig. 4c), suggesting that on the *03g* locus JMJ703 might be more active in differentiated cells than in callus. In *jmj703/chr729* double mutant leaves, *03g* expression was increased back to the level of regenerated wild type plants, but there was no clear difference for *03g* expression in callus between *chr729* and the double mutant (Fig. 4c,d). This observation supported the hypothesis that *JMJ703* preferentially functioned in differentiated tissues and suggested that higher H3K4me3 in *jmj703* mutant might compensate the loss of H3K4me3 for *03g* expression in *chr729* plants. CHR729 and JMJ703 might be functional antagonists for H3K4me3 in a subset of genes, as *jmj703* mutation resulted in elevated H3K4me3 in many loci^22^, while loss-of-function of *CHR729* leads to decrease of H3K4me3 preferentially from under-expressed genes^13^. Comparison of the two sets of data revealed that half of the genes that showed higher H3K4me3 in *jmj703* were among those with decreased H3K4me3 in *chr729* (Supplementary Table 1). We tested some of the loci and found that the decrease of H3K4me3 in *chr729* mutant was partially or fully recovered in the *jmj703*/*chr729* double mutant (Supplementary Figure 4). This suggests that the two proteins may have antagonistic function in H3K4me3 of a subset of loci in the genome.

### SDG711-mediated H3K27me3 is sufficient to stably repress the epiallele

ChIP assay with anti-SDG711, prepared with *E. coli*-produced SDG711 protein and previously tested^14^, revealed a direct association of SDG711 to the *03g* locus (Fig. 4e). A lower level of SDG711-binding detected in regenerated plants (crDJ) might be due to *03g* activation that might consequently inhibit SDG711 association. H3K27me3 levels on *03g* were maintained in *sdg711* and *Ubi: SDG711* and were reduced in *SDG711* RNAi plants compared to wild type (Fig. 4f). Likely, *SDG711*-mediated maintenance of H3K27me3 was sufficient to mediate *03g* stable repression. Meanwhile, in *sdg711* plants *03g* displayed similar levels of DNA methylation as in wild type (Fig. 4b), suggesting that either the maintenance of H3K27me3 or the association of SDG711 to the locus prevented DNA methylation loss from the locus in regenerated plant. Consistent with the repressive state of *03g,* the H3K4me3 levels in *sdg711* and *Ubi: SDG711* plants were similar to that in wild type (Fig. 4g).

The mechanism that causes DNA hypomethylation in regenerated plants which occurs preferentially at promoter regions is not known^11^. As the T-DNA mutant line of *DNG701*^17^, which encodes a rice DNA demethylase, showed *03g* expression (Table 1), it is unlikely that this demethylase activity was involved in the DNA methylation loss from the locus during callus culture. Previous results have indicated that siRNAs play an important role in the recovery of DNA methylation loss from a majority of genomic regions over generations in Arabidopsis^4^, and that stable DNA hypomethylation in regenerated rice plants may be due to loss of relevant siRNA^11^. The present data suggest that deposition of H3K4me3 during callus-induced gene activation also plays a role in maintaining stable DNA hypomethylation and expression in regenerated plants. This is consistent with previous results showing that H3K4me3 can prevent DNA remethylation^29^,^30^,^31^,^32^. The recovery of *03g* DNA methylation by over-expression of H3K4me3 demethylase gene *JMJ703* corroborated the observations that mutation of *Arabidopsis* H3K4 demethylase genes reduces DNA methylation in genomic regions that show coincidental increases in H3K4me2/me3^30^. The function of CHR729 in maintaining *03g* H3K4me3 likely contributed to the maintenance of active state of the locus. Therefore, CHR729/JMJ703-regulated H3K4me3 homeostasis may provide a mechanism to stabilize active epialleles that could be generated by epigenomic variations in plants, although it is not excluded that an independent function of CHR729 might be involved. In addition, our data show that stable *03g* silencing was not only mediated by DNA methylation but also by SDG711-mediated H3K27me3, suggesting that SDG711 may be involved in transgenerational silencing of gene expression. Taken together, the data in this work suggest that variation in expression levels of histone methylation regulatory genes may contribute to stable transmission or resetting of epigenetic variation of gene expression in crop plants.

## Methods

### Rice materials

Rice (*Oryza sativa*) cultivated varieties ZH11 (Zhonghua11), MH63 (Minghui63), DJ (Dongjin), HY (Hwayoung), SSBM (Shi Shou Bai Mao) were used in this study. Callus culture and plant regeneration were performed as described previously^33^. Briefly, rice seeds were sterilized and placed on callus induction media for 30 days, then primary callus was transferred to subculture media. After two weeks, callus was used for transformation or transferred to regeneration media. Primary plants regenerated from callus were transferred to rooting media and transferred to field or greenhouse after 1 week. T-DNA mutants, RNAi and over-expression plants were generated by *Agrobacterium*-mediated transformation using standard callus culture, transformation, selection, and regeneration procedures^33^.

### Gene expression analysis

RNA was extracted from rice leaves or callus with TRIzol reagent (15596-026, Life Technology) and reverse-transcribed with SSIII (18080-044, Life Technology). Synthesized first strand cDNA was used as template for regular PCR amplification or real-time PCR (ABI7500).

### 5-azacytidine treatment

Sterilized rice seeds were placed on 1/2 MS medium containing 50 mg/L 5-aza-2′-deoxycytidine (A3656, Sigma) and grown under dark condition for 1 month. Treated seedlings (aerial parts) were then harvested and pooled for further analysis or transferred to normal condition for propagation.

### DNA methylation assay

Genomic DNA was extracted from rice leaf and callus. For bisulfite sequencing, 1 μg DNA was treated with bisulfite salt and recovered using EpiTect Bisulfite Kit (Qiagen, 59104). PCR fragments were cloned into T Easy Vector (Promega, A1360) for sequencing. Sequences were analyzed using online software (http://katahdin.mssm.edu/kismeth/revpage.pl). At least 20 clones were sequenced for each. For Enzyme Digestion PCR analysis, 500 ng DNA was treated with 20U *Hae*III, *Hpa*II and *Msp*I for 4 hours in a 20 μl reaction volume, respectively. Digested DNA (1 μl) was used as PCR template. DNA fragments without recognition sites of those enzymes were amplified as DNA loading control.

### ChIP assay

ChIP analysis was performed as previous described^23^. Briefly, chromatin isolated from 2 g rice leaf or 1 g callus was incubated with antibody coated beads (Life technology, 10001D) overnight. After wash and elution, products were reverse crosslinked. Then the products were treated with protease K (Takara, 9034), recovered, and used as template for real-time PCR with primers listed in Supplementary Table 2. Antibodies for histone modifications are anti-H3K4me3 (ab8580, Abcam), anti-H3K27me3 (07–449, Millipore) and anti-FLAG (F3165, Sigma) antibodies respectively. Antibody of SDG711 was produced by immunizing rabbits with *E. coli* produced full-length SDG711 protein^14^.

### Accession Numbers

*DMT702* (Os03g58400), *DMT703* (Os05g13790)*, DMT706* (Os03g02010), and *DMT707* (Os07g08500), *DNG701* (Os05g37350), *SDG711* (Os06g16390)*, SDG721* (Os01g11950), *SDG723* (Os09g04890) and *SDG728* (Os05g41170), *JMJ703* (Os05g10770), *JMJ705* (Os01g67970), *JMJ706* (Os10g42690), *JMJ716* (Os03g22540), *CHR729* (Os07g31450), *OsDDM1* (Os09g27060), *OsLHP1* (Os10g17770).

## Additional Information

**How to cite this article**: Chen, X. *et al.* Histone H3K4me3 and H3K27me3 regulatory genes control stable transmission of an epimutation in rice. *Sci. Rep.* **5**, 13251; doi: 10.1038/srep13251 (2015).

## Supplementary Material

Supplemental Figures

## Figures and Tables

**Table 1:** Detection of *03g* expression in T-DNA mutants and transgenic plants of rice chromatin regulators.

	Name	*03g*expression	References
DNA methyltransferase	*DMT702* T-DNA mutant	Yes	*
	*DMT703* RNAi	Yes	*
	*DMT706* over-expression	Yes	34,*
	*DMT707* RNAi	Yes	*
DNA glycosylase	*DNG701* T-DNA mutant	Yes	17
Histone methylation	*SDG711* gain of function T-DNA	No	14
	*SDG711* over-expression	No	14
	*SDG711* RNAi	Yes	14
	*SDG721* RNAi	Yes	*
	*SDG723* RNAi	Yes	*
	*SDG728* over-expression	Yes	20
Histone demethylase	*JMJ703* T-DNA mutant	Yes	21,22
	*JMJ703* over-expression	No	21,22
	*JMJ705* T-DNA mutant	Yes	24
	*JMJ705* over-expression	Yes	24
	*JMJ706* T-DNA mutant	Yes	23
	*JMJ716* T-DNA mutant	Yes	*
Chromatin remodeler	*DDM1b* T-DNA mutant	Yes	25
	*CHR729* T-DNA mutant	No	13
	*LHP1* RNAi	Yes	*
	*LHP1* over-expression	Yes	*

^*^characterization of the mutant, over-expression or RNAi plants are shown in Supplementary Figure 4.
