# Supplementary material for: Histone H3K4me3 and H3K27me3 regulatory genes control stable transmission of an epimutation in rice
Source: Sci Rep. 2015 Aug 19;5:13251. doi: 10.1038/srep13251 (PMC4541256; doi:10.1038/srep13251)
Supplement: Supplemental Figures [file srep13251-s1.pdf]

Histone H3K4me3 and H3K27me3 regulatory genes control stable transmission of an epimutation in rice

Xiangsong Chen[1], Xiaoyun Liu[1,2], Yu Zhao[1], Dao-Xiu Zhou[1,3]*

[1]National Key Laboratory of Crop Genetic Improvement, Huazhong Agricultural University, 430056 Wuhan, China

[2]Institute for Interdisciplinary Scientific Research, Jianghan University, 430070 Wuhan, China

[3]Institute Plant Science Paris-Saclay (IPS2), Université Paris-sud 11, 91405 Orsay, France

*Corresponding author (dao-xiu.zhou@u-psud.fr)

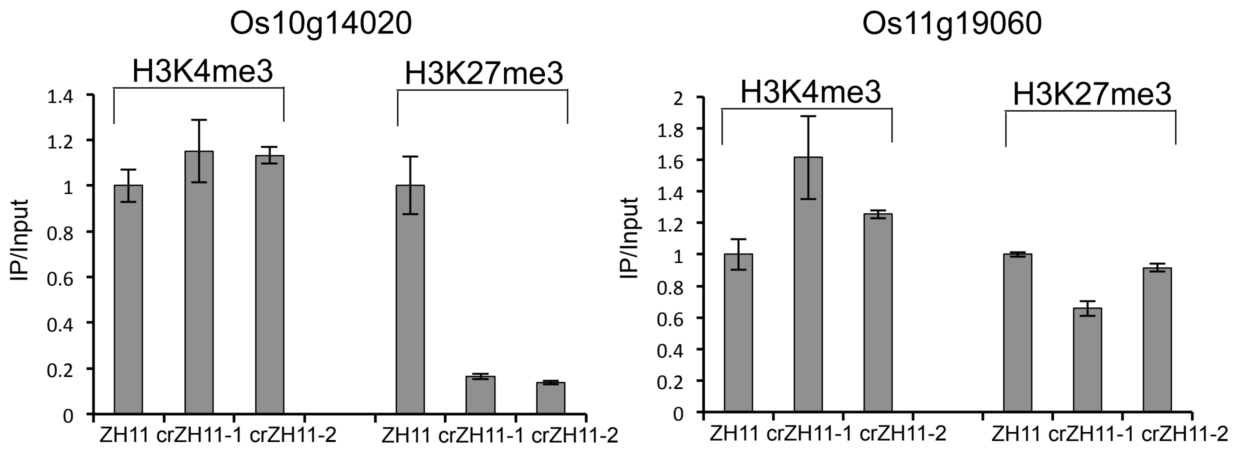

Supplementary figure 1. H3k4me3 and H3K27me3 at Os10g14020 and Os11g19060 in wild type (ZH11) and two callus regenerated plants (crZH11-1/-2).
Primers are around transcription start site of these two loci. All samples are normalized with ZH11.

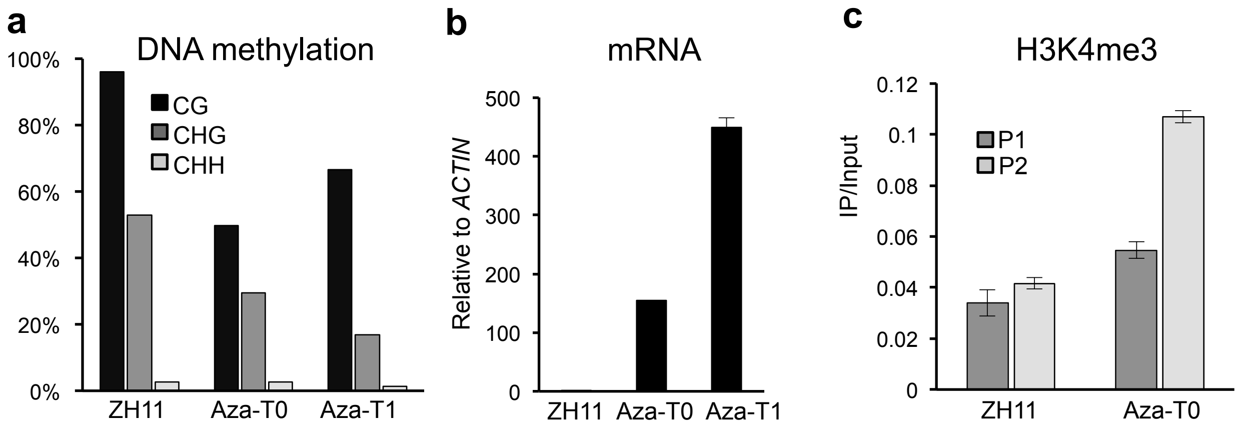

Supplementary figure 2. Inhibition of DNA methylation induces stable 03g activation.

Treatment with DNA methylation inhibitor 5-azacytidine led to 03g DNA demethylation (a), gene expression (b) and increased H3K4me3 (c). Two week-old treated seedlings (aerial part) were pooled for DNA methylation analysis. The expression level is normalized with *ACTIN* and then set as 1 in ZH11. Primers are the same as P1 and P2 indicated in Figure 2a. At least 3 biological replicated were performed; only one set of data are shown. Error bars represent technical repeats of qPCR.

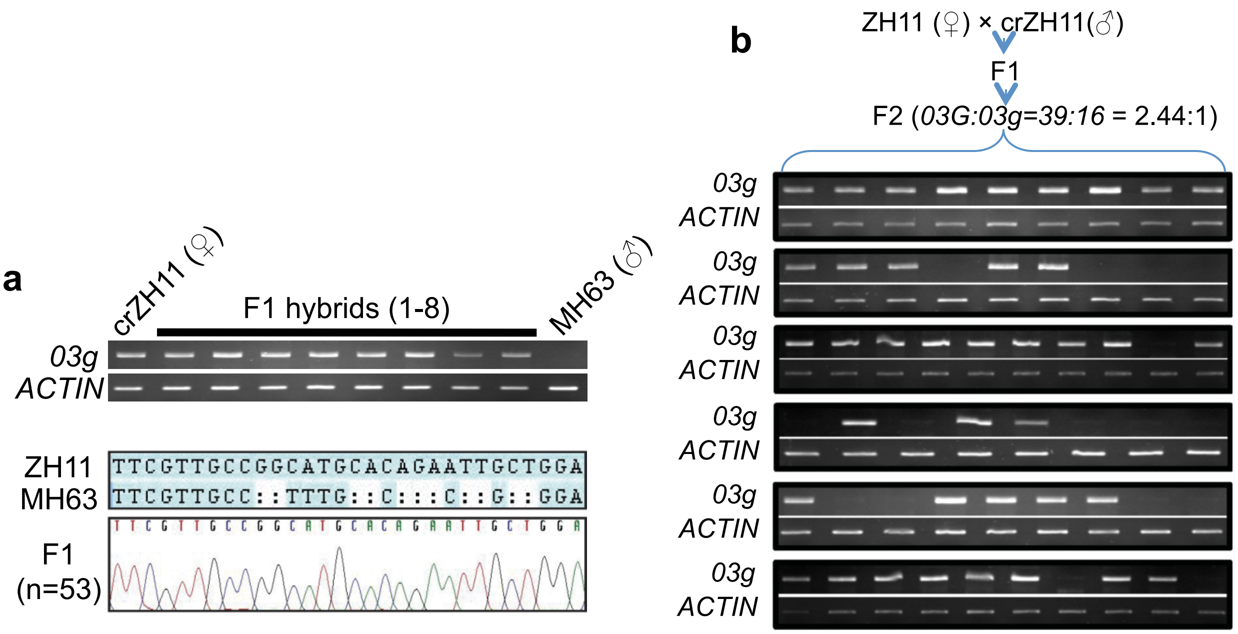

Supplementary figure 3. *03g* expression is not affected by its hypermethylated allele in F1 hybrids.

(a) Upper: *03g* was active in F1 hybrids of crosses between normal wild type MH63 (male) and regenerated ZH11 (female). Lower: *03g* transcripts were reverse-transcribed and sequenced. All of the sequenced transcripts from F1 hybrids were from ZH11. (b) Segregation of *03g* expression (analyzed by RT-PCR) in F2 population of crosses between normal wild type ZH11 and regenerated ZH11 (crZH11).

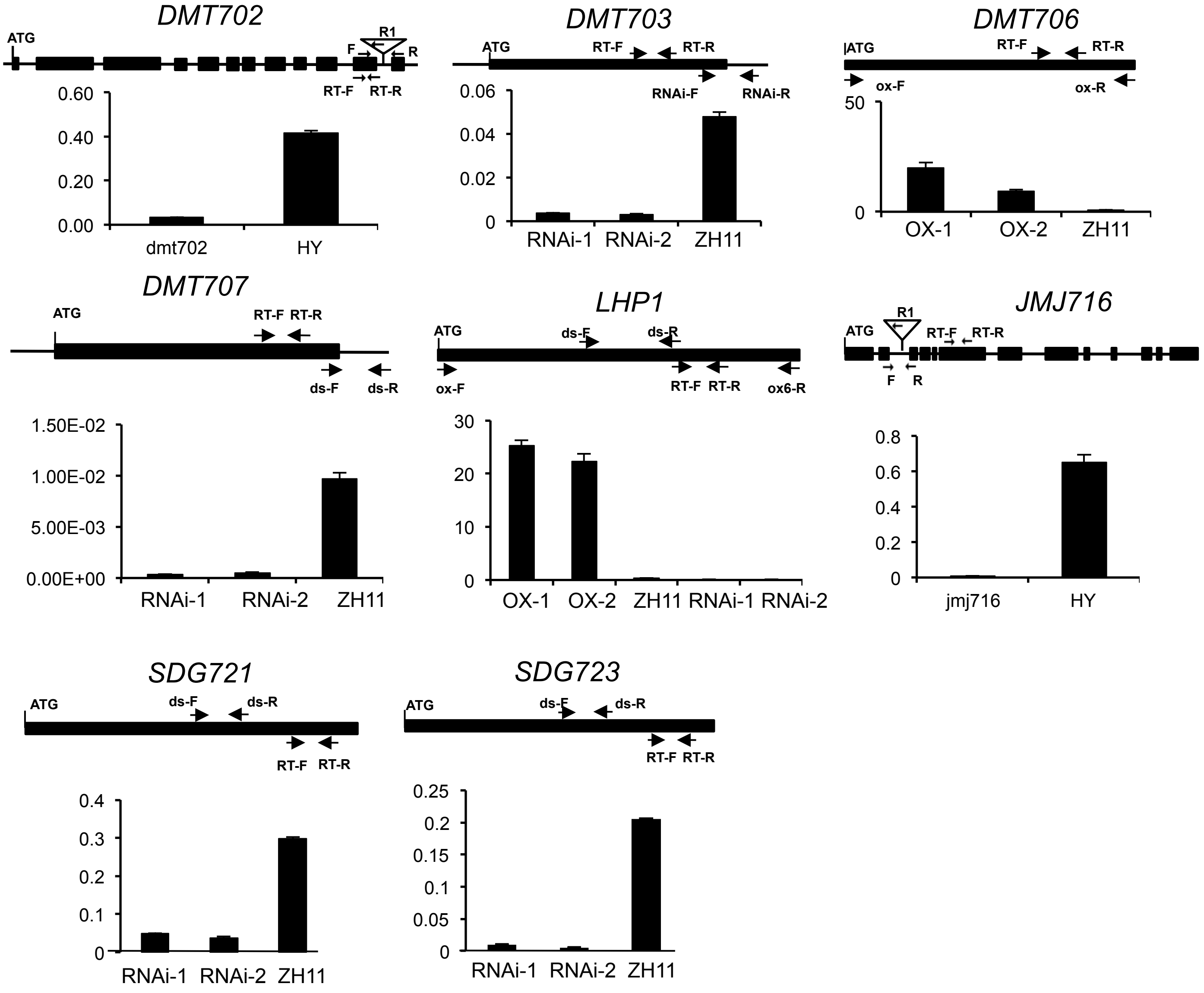

Supplementary figure 4. Characterization of rice chromatin regulator gene T-DNA insertion, RNAi and over-expression plants.

Relative positions on the genes of the primers used for PCR amplification for transgenic vector construction and for RT-PCR are indicated by small arrows. T-DNA insertions are indicated by open triangles. Relative transcript levels (to *ACTIN* mRNA) of the T-DNA lines and two transgenic lines per construct were analyzed to compare with the respective wild type (ZH11, or HY).

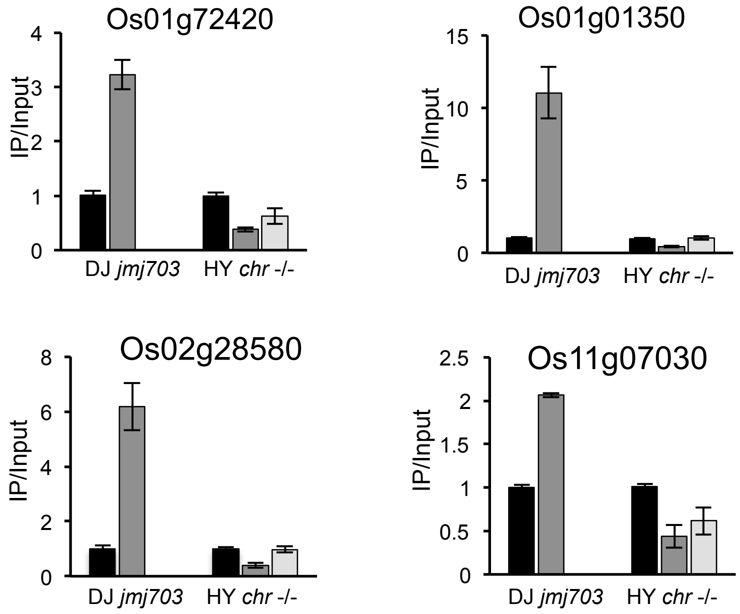

Supplementary figure 5. ChIP analysis of H3K4me3 in *jmj703, chr729* and their double mutant on representative loci. Four genes from Table S1 were analyzed for H3K4me3 in *jmj703*, *chr729* single and double (-/-) mutants. Wild type (DJ and HY) was set as 1.

Table S1. Relative levels of H3K4me3 in chr729 mutant compared to wild type (HY) of genes that display increased H3K4me3 in *jmj703* mutants

**Locus**	**Functiondescription**	***chr729*/HY**
LOC_Os01g01400	expressed protein	0
LOC_Os02g28580	expressed protein	0
LOC_Os03g05530	nodulin, putative, expressed	0
LOC_Os03g14420	cytochrome P450, putative, expressed	0
LOC_Os03g31730	expressed protein	0
LOC_Os12g08760	carboxyvinyl-carboxyphosphonatephosphorylmutase, putative, expressed	0
LOC_Os01g01350	SNF7 domain containing protein, putative, expressed	0.442
LOC_Os03g51600	tubulin/FtsZ domain containing protein, putative, expressed	0.504
LOC_Os01g72420	C2 domain containing protein, putative, expressed	0.588
LOC_Os06g41010	zinc finger A20 and AN1 domain-containing stress-associated protein, putative, expressed	0.598
LOC_Os04g10450	hypothetical protein	0.603
LOC_Os11g07030	integrator complex subunit 3, putative, expressed	0.618
LOC_Os02g52730	ferredoxin--nitrite reductase, putative, expressed	0.625
LOC_Os01g18650	transposon protein, putative, unclassified	0.629
LOC_Os01g34050	expressed protein	0.717
LOC_Os01g49950	transposon protein, putative, unclassified	0.676
LOC_Os03g57160	zinc ion binding protein, putative, expressed	0.688
LOC_Os06g19370	cadmium tolerance factor, putative, expressed	0.718
LOC_Os06g15370	peptide transporter PTR2, putative, expressed	0.723
LOC_Os07g39120	hypothetical protein	0.764
LOC_Os02g42910	expressed protein	0.956

Table S2. Primers used in this study

Primers for vector construction	
dsDMT703-F	GGGACTAGTGGTACCAGTATGGTGGGATGAGGTAGTGG
dsDMT703-R	GGGGAGCTCGGATCCTGGCAACAAGAAGAGGAGCA
dsDMT707-F	GGGACTAGTGGTACCGCGTCAGTAGGTGCTCAGAGTC
dsDMT707-R	GGGGAGCTCGGATCCCTGAATCAAGGGTATCAATCACA
oxDMT706-F	TTACGAACGATAGCCGGTACATGGTGGACTGGGCTTCAGATAG
oxDMT706-R	TTTGTAATCGGATCCGGTAC CTAGACTGCTGACATGGTAGCCT
dsLHP1-F	ACTAGTGGTACCGGCTGCAGTGAATGACGATG
dsLHP1-R	GAGCTCGGATCCTCACTTGGCGGGGTGCTTGG
oxLHP1-F	GGTACCCCGATTCCGATGGAGTTCTG
oxSDG711-F	TTACGAACGATAGCCGGTACCTGATGGCTGGCGATTCC
oxSDG711-R	TTTGTAATCGGATCCGGTACCGAATGGCAGGAAAGTTTTCC
dsSDG711-F	AGAACTAGTGGTACCAGAAGCTGACAAGCGTGGAAAG
dsSDG711-F	AGAGAGCTCGGATCCCGACAGGTGCAGCAGTTACAG
dsSDG721-R	GGGGAGCTCGGATCCCAATCTTTCTTCTCCCTGTGATA
dsSDG723-F	GGGACTAGTGGTACCGACAAAATCCACCTCATGTTACT
dsSDG723-R	GGGGAGCTCGGATCCTTGACTTTCCAAAAGCTAGTCTC
Primers for T-DNA detection	
dmt702-F	CCCCCAGTGCTATGATTCTC
dmt702-R	TGTGCTACAGTACGGAAGCG
Korea-R	TTGGGGTTTCTACAGGACGTAAC
jmj716-F	TGCATCTCGAGATGGTATGC
jmj716-R	CTGCAGTTACAAATGCCACG
SDG711-F	GCCTTCCGCCCTCCT
SDG711-R	CGGTCCGATGTGATTTTCTT
SDG711-RB2	GGACCTGCATATAACCTGCA
Primers for quantitative RT-PCR	
Actin-QF	TGAAGATCAAGGTGGTGGCAC
Actin-QR	TGCTGGACCCGACTCATCATA
ActinRT-F	TCCATCTTGGCATCTCTCAG
ActinRT-R	GTACCCGCATCAGGCATCTG
03g-QF	GGATCTCCGACGATTGTATTCACT
03g-QR	CGTTTGTTGTTATCAATTTCTTGCA
03gRT-F	TCAAGCTTTGAGGTCGCTGGCCA
03gRT-R	GTTATCAATTTCTTGCAAGGTGA
DMT702-QF	TGCCCAACACAGCCAAAAG
DMT702-QR	TTCCCCATTGGCTGAGGAT
DMT703-QF	ACACCCTTGTCAGAAGGCACA
DMT703-QR	TGGCAACAAGAAGAGGAGCAG
DMT706-QF	AGAAGGTTTGGCGGTTTTGAC

DMT706-QR	AATCGTAGAAGAGCGCGGAGT
DMT707-QF	CAGAGCAAGCACAGGCAGATT
DMT707-QR	GCACCTACTGACGCTTTGCAT
OsLHP1-QF	CGCTTTTTGCACCTTTCTCAG
OsLHP1-QR	ACTTTGCCATTTGCCCTGG
JMJ716-QF	TTTGATTATCACCGACACTGCC
JMJ716-QR	TCTACCACACGACCTTCAGCAT
SDG711-QF	AGGAGCCAACATCGGACAAT
SDG711-QR	TGAGGAAACCCTTGCAGTTGA
SDG721-QF	GTTAGACGTAGTGTTGCA
SDG721-QR	CGAGTGATTAATTAGGCGGG
SDG723-QF	GCAAAGCGGGATATAAATC
SDG723-QR	CCCTTATTTTGGCTGATTGCC
Primers for ChIP analysis	
Actin-CF	TGCGTCAGGAATTCAGAACCA
Actin-CR	AGCACCACGAACCTTGACCAT
03gP1-CF	GTGAATAGGAGCTGCATGAAATCC
03gP1-CR	ACGTTTTTCACCAACTGGGC
03gP2-CF	CGAGATTGCCACACGGTAAGA
03gP2-CR	AGCCCAGGACTCGGTAGATTC
03gP3-CF	CACTCGATCTCCCCTTTTGC
03gP3-CR	AAACAACGTGGGGTGTGAGC
03gP4-CF	CGCCGGTGACGGGAG
03gP4-CR	ACTATACGAAGGAGACGCAATTGA
